# Diagnostic value of serum versus plasma phospho-tau for Alzheimer’s disease

**DOI:** 10.1186/s13195-022-01011-w

**Published:** 2022-05-11

**Authors:** Przemysław R. Kac, Fernando Gonzalez-Ortiz, Joel Simrén, Nele Dewit, Eugeen Vanmechelen, Henrik Zetterberg, Kaj Blennow, Nicholas J. Ashton, Thomas K. Karikari

**Affiliations:** 1 Department of Psychiatry and Neurochemistry, Institute of Neuroscience and Physiology, The Sahlgrenska Academy, University of Gothenburg, Gothenburg, Sweden; 2 Clinical Neurochemistry Laboratory, Sahlgrenska University Hospital, Mölndal, Sweden; 3 ADx NeuroSciences, Technologiepark 94, Ghent, Belgium; 4 Department of Neurodegenerative Disease, UCL Institute of Neurology, London, UK; 5 UK Dementia Research Institute at UCL, London, UK; 6 Hong Kong Center for Neurodegenerative Diseases, Hong Kong, China; 7 Wallenberg Centre for Molecular and Translational Medicine, University of Gothenburg, Gothenburg, Sweden; 8 King’s College London, Institute of Psychiatry, Psychology & Neuroscience, Maurice Wohl Clinical Neuroscience Institute, London, UK; 9 NIHR Biomedical Research Centre for Mental Health & Biomedical Research Unit for Dementia at South London & Maudsley NHS Foundation, London, UK; 10 Department of Psychiatry, University of Pittsburgh, Pittsburgh, PA USA

**Keywords:** Alzheimer’s disease, Blood biomarkers, Phosphorylated tau, Ethylenediaminetetraacetic acid, Plasma, Serum, p-tau231, p-tau181, Cerebrospinal fluid

## Abstract

**Background:**

Blood phosphorylated tau (p-tau) forms are promising Alzheimer’s disease (AD) biomarkers, but validation in matrices other than ethylenediaminetetraacetic acid (EDTA) plasma is limited. Firstly, we assessed the diagnostic potential of p-tau231 and p-tau181 in paired plasma and serum samples. Secondly, we compared serum and cerebrospinal fluid (CSF) samples from biomarker-positive AD and biomarker-negative control participants.

**Methods:**

We studied three independent cohorts (*n*=115 total): cohorts 1 and 2 included individuals with paired plasma and serum, while cohort 3 included paired serum and CSF. Blood-based p-tau231 and p-tau181 were measured using in-house or commercial single molecule array (Simoa) methods.

**Results:**

Serum and plasma p-tau231 and p-tau181 were two- to three-fold increased in biomarker-positive AD versus biomarker-negative controls (*P*≤0.0008). Serum p-tau231 separated diagnostic groups with area under the curve (AUC) of 82.2% (cohort 3) to 88.2% (cohort 1) compared with 90.2% (cohort 1) for plasma. Similarly, p-tau181 showed AUC of 89.6% (cohort 1) to 89.8% (cohort 3) in serum versus 85.4% in plasma (cohort 1). P-tau231 and p-tau181 correlated slightly better in serum (rho=0.92 for cohort 1, 0.93 for cohort 3) than in plasma (rho=0.88, cohort 1). Within-individual p-tau181 (Quanterix) and p-tau231 concentrations were twice higher in plasma versus serum, but p-tau181 (in-house, Gothenburg) levels were not statistically different. Bland-Altman plots revealed that the relative difference between serum/plasma was larger in the lower range. P-tau levels in paired plasma and serum correlated strongly with each other (rho=0.75–0.93) as well as with CSF Aβ_42_ (rho= −0.56 to −0.59), p-tau and total-tau (rho=0.53–0.73). Based on the results, it seems possible that serum p-tau reflects the same pool of brain-secreted p-tau as in CSF; we estimated that less than 2% of CSF p-tau is found in serum, being same for both controls and AD.

**Conclusions:**

Comparable diagnostic performances and strong correlations between serum versus plasma pairs suggest that p-tau analyses can be expanded to research cohorts and hospital systems that prefer serum to other blood matrices. However, absolute biomarker concentrations may not be interchangeable, indicating that plasma and serum samples should be used independently. These results should be validated in independent cohorts.

## Background

Recent studies have shown blood phosphorylated tau (p-tau) forms to be reliable biomarkers in supporting a diagnosis of Alzheimer’s disease (AD) [1–5] and in screening for individuals with biomarker evidence of the disease in the absence of cognitive impairment [6–10]. Blood-based p-tau biomarkers increase according to amyloid beta (Aβ) pathology and disease severity, associate well with established cerebrospinal fluid (CSF) and neuroimaging biomarkers, and differentiate biomarker-positive AD dementia from other dementias as well as Aβ-negative controls [1, 2, 5, 6, 11]. Importantly, blood p-tau is highly accurate at detecting brain amyloidosis and in predicting those who will progress to cognitive impairment and neurodegeneration, often at similar magnitude as CSF p-tau [2, 6, 8, 12–16]. These findings support the integration of blood p-tau analyses into routine clinical assessments and population screening programs to identify biological evidence of AD, especially in the face of the recent approval of the anti-amyloid drug aducanumab (Aduhelm®) and the ongoing consideration of other anti-amyloid therapies by the US Food and Drug Administration (FDA) and equivalent regulatory agencies elsewhere [17–21].

Despite the rapid progress, several analytical hurdles need to be addressed to allow for large-scale adoption of blood p-tau in clinical and research settings. For example, recent head-to-head comparison studies have demonstrated that blood p-tau biomarkers from independent research laboratories and biotechnology/pharmaceutical companies show high inter-assay correlations as well as analytical and diagnostic robustness for clinical use [22, 23]. However, these studies have been limited to the use of blood processed into ethylenediaminetetraacetic acid (EDTA) plasma [22, 23]. Despite EDTA plasma being the most commonly used blood matrix type in the dementia biomarker field, it is unclear if other matrices are equally viable given previous reports of large matrix-dependent deviations in biomarker concentrations [24–26]. With blood p-tau already being included in anti-amyloid clinical trial programs and with planned diagnostic applications expected in several clinics, it is vital to ensure the widespread use of these biomarkers in multiple settings including research and medical centers that preferably process blood into other matrix types [18–20, 27, 28].

So far, only p-tau181 has been shown to be measurable in paired samples and in matrices other than EDTA plasma [1, 24, 26, 29]. Despite strong inter-matrix correlations, p-tau181 concentrations varied significantly between paired samples in different matrices [24, 26]. This was also true for total-tau, suggesting that variable matrix-dependent concentrations may be common to tau biomarkers and not just to p-tau [24, 26]. These results also point to a need for thorough verification of tau-based biomarkers in non-EDTA plasma matrices prior to clinical use.

Blood biomarker verification in serum is essential given its widespread use in clinical settings. However, direct comparison of the diagnostic performances of different p-tau forms in serum is limited. In this proof-of-concept study, we investigated if p-tau231 and p-tau181 can be reliably measured in serum versus paired plasma samples to distinguish biomarker-positive AD cases from biomarker-negative controls, as previously shown for plasma and CSF in multiple independent cohorts [1, 5, 6, 9, 10, 13, 30–34]. We then compared inter-matrix agreements between p-tau measures, and further validated the serum performance by evaluating associations with paired CSF samples.

## Methods

### Study participants

We studied three independent cohorts of *n* = 33, 47, and 35 individuals respectively (*n* = 115 total). Participants in cohort 1 and cohort 3 were pre-classified as biomarker-positive AD or biomarker-negative controls according to their neurochemical CSF biomarker profiles. Cohorts 1 and 3 included 18 and 19 biomarker-positive AD participants respectively. There were 15 and 16 biomarker-negative controls from cohort 1 and cohort 3 respectively. Both cohorts were recruited from the Sahlgrenska University Hospital, Mölndal, Sweden. Cohort 2 included a set of paired plasma and serum samples collected from apparently healthy volunteers, ranging in age from 22 to 69, collected with informed consent and in accordance with the Declaration of Helsinki. EDTA-plasma and serum were collected in February and September 2021, according to state-of-the-art recommendations [26]. Every blood draw was tested for infectious disease (HIV, HCV), and total protein, albumin, and hemoglobin concentrations were within the normal range for all individuals.

The AD participants in cohort 1 and cohort 3 were clinically assessed for suspected AD. The control participants had their core CSF biomarkers in normal ranges. CSF Aβ_42_, p-tau181, and total-tau were measured with the INNOTEST® β-AMYLOID (1-42), PHOSPHO-TAU (181P), and hTAU Ag immunoassays as previously described [35], and the results were used to categorize participants into biomarker-negative controls and biomarker-positive AD. In both cohorts, biomarker positivity was established according to the following cut-offs: Aβ_42_ <530 pg/ml, total-tau >350 ng/ml, and p-tau181 >60 pg/ml.

### Blood and CSF sample collection and biochemical measurements

To study p-tau in plasma and serum from the same individuals, we collected whole blood by venipuncture in cohort 1 and processed them concurrently into EDTA plasma and serum. We transferred 5-ml aliquots from the same blood draw into Vacuette® tubes (Greiner Bio-One) for serum and EDTA plasma (catalogue numbers 456234 and 456243 respectively). Samples were processed following standard clinical chemistry procedures. In brief, the tubes were centrifuged within 2 h of collection at 2000×*g* for 10 min and stored at −80°C until use. Sample collection for cohort 2 followed procedures according to the latest recommendations described recently [26]. In cohort 3, serum samples were processed as described for cohort 1. CSF collection by lumbar puncture followed standard clinical practices [36].

Prior to biomarker analysis, blood samples stored at −80°C were thawed, vortexed, and centrifuged at 4000×*g* for 10 min. For cohort 1 and cohort 3, p-tau231 and p-tau181 were measured in plasma (two-fold diluted), serum (two-fold diluted), and CSF (30-fold diluted) using validated in-house single molecule array (Simoa) methods [1, 5]. Briefly, the p-tau-specific mouse monoclonal antibodies ADx253 (ADx NeuroSciences) and AT270 (Thermo Fisher) were used as capture for p-tau231 and p-tau181 respectively. Both assays used the N-terminal-targeting Tau12 (BioLegend) antibody for detection. Quality control samples were analyzed in duplicates at the start and the end of each plate to assess precision. The within- and between-run variations were respectively 0.6–12% and 2.1–12% for p-tau231, and 0.2–5.7% and 2.8–5.7% for p-tau181 in cohort 1. For cohort 3, the within- and between-run variations were respectively 2.8–6.4% and 3.1–6.4% for p-tau231, and 1.7–5.3% and 2.5–5.3% for p-tau181. For serum p-tau231, four samples in cohort-1 and three samples in cohort-3 measured below the limit of detection. All p-tau181 measures were above the limit of detection. For each assay, identical batches of reagents were used for all matrix types to enable comparison of results. These measurements were performed at the Clinical Neurochemistry Laboratory, Sahlgrenska University Hospital, Mölndal, Sweden.

Paired plasma and serum samples from cohort 2 were measured using the commercial Simoa^TM^ pTau-181 Advantage V2 kit available from Quanterix (Billerica, MA, USA). We used this method according to the manufacturer’s instructions to measure p-tau181 in paired plasma and serum samples from *n*=47 individuals. All 94 quantifications were performed in duplicate in one run and variability of the two run controls and its assigned values were in the expected ranges (control 1 range 2.5–3.7 pg/ml with a within-run variation of 3.5% for a concentration of 2.9 pg/ml; control 2 with expected concentration range from 92 to 138 pg/ml had a measured concentration of 117.7 pg/ml with within-run variation of 0.7%). For serum p-tau181, one sample measured below the limit of detection. These measurements were performed at ADx NeuroSciences, Ghent, Belgium.

### Statistical analyses

Statistical analyses were performed with Prism version 9 (GraphPad, San Diego, CA, USA). Non-parametric tests were used for non-normally distributed data. Continuous and categorical variables were evaluated with Spearman correlation and *χ*^2^ test respectively. Mann-Whitney test was used for group comparisons, and area under the receiver operating characteristics curve (AUC) to estimate diagnostic performance. Fold changes were calculated by dividing p-tau concentrations by the mean data for the control group. Bland-Altman plots were made to assess agreement of the p-tau measurements in paired serum and plasma [37]. The 95% limits of agreement (LOA) were estimated by calculating the mean ± 2 standard deviations. For all statistical analyses, significance was set at two-sided *p*<0.05.

## Results

### Cohort characteristics

Cohort 1 included 15 females and 18 males, with a distribution of 33.3% and 55.5% females among the controls and AD groups, respectively. The mean age was 67.3±9.1 years for controls and 77.4±5.8 years for AD (Mann Whitney U=48, *P=*0.0011). Cohort 2 included 47 individuals (27 female, 20 male) between the ages of 20 and 69 years. In cohort 3, there were 20 females and 15 males with comparable distributions between groups. The mean age was 66.6±12.3 years, without inter-group differences; 65.5±15.3 years for controls and 67.7±8.6 years for AD (Mann Whitney *U*=199.5, *P=*0.9947). The demographic characteristics are summarized in Table 1.

**Table 1:** Demographic characteristics of the study participants

	Cohort 1		Cohort 2	Cohort 3	
	Biomarker-negative controls	Biomarker-positive AD	Control individuals	Biomarker-negative controls	Biomarker-positive AD
Sample size	15	18	47	16	19
Age, y	67.3 ± 9.1	77.4 ± 5.8^a^	45,9 ± 15,2	65.5±15.3	67.7±8.6
Gender, F, *n (%)*	5/15 (33.3%)	10/18 (55.5%)	27/47 (57.4%)	10/16 (62.5%)	10/19 (52.6%)
CSF Aβ42, pg/ml	1002.0 ± 286.6	450.9 ± 67.9^a^	N/A	877.2 ± 160.7	499.4 ± 87.0^a^
CSF total-tau, pg/ml	262.0 ± 81.0	773.7 ± 406.9^a^	N/A	209.2 ± 61.2	577.3 ± 136.1^a^
CSF p-tau181 (Innotest), pg/ml	42.5 ± 12.7	91.2 ± 26.2^a^	N/A	28.4 ± 8.0	88.9 ± 23.7^a^
CSF p-tau181 (Simoa), pg/ml	N/A	N/A	N/A	176.4 ± 57.3	895.8 ± 350.9^a^
CSF p-tau231 (Simoa), pg/ml	N/A	N/A	N/A	154.4 ± 49.4	777.9 ± 260.8^a^
Plasma p-tau231, pg/ml	6.5 ± 3.4	13.4 ± 5.2^a^	N/A	N/A	N/A
Plasma p-tau181, pg/ml	5.8 ± 2.8	10.4 ± 4.1^a^	1.1 ± 0.4	N/A	N/A
Serum p-tau231, pg/ml	2.5 ± 2.3	6.4 ± 2.7^a^	N/A	1.1 ± 1.1	3.8 ± 4.3^a^
Serum p-tau181, pg/ml	4.5 ± 2.0	9.3 ± 3.4^a^	0.6 ± 0.3	2.5 ± 2.2	10.1 ± 10.4^a^

Biomarker and age differences were tested using Mann-Whitney test

Biomarker concentrations are shown as mean ± standard deviation (SD)

Note that p-tau measurements were performed with different methods in cohorts 1 and 3 (validated in-house assays) vs. cohort 2 (commercial kit from Quanterix)

*N/A*, not available

^a^Significant difference compared with controls

### Diagnostic performance of serum vs. plasma p-tau

**Fig. 1:**
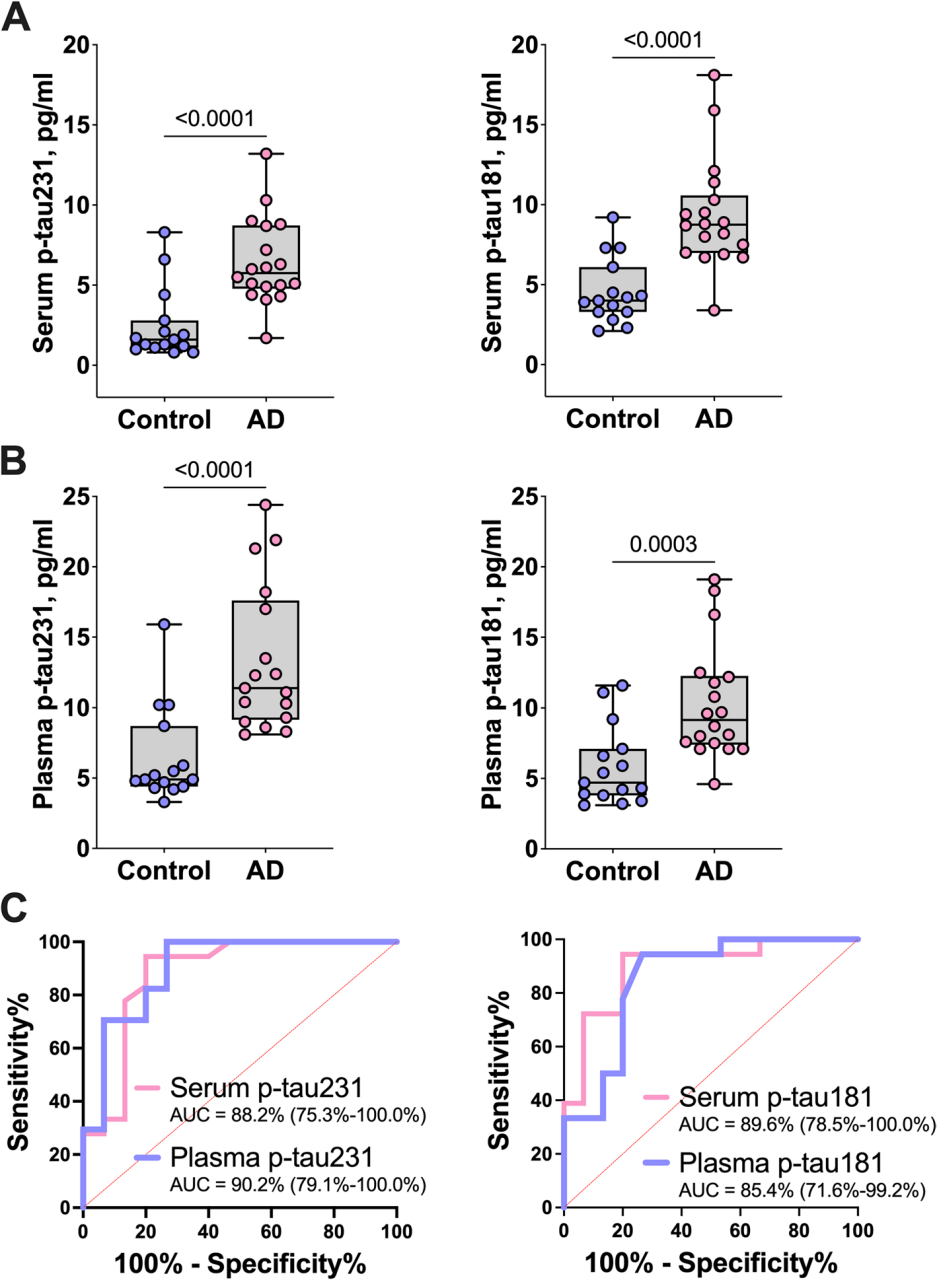
Profile of serum vs*.* plasma p-tau in paired samples from neurochemically defined biomarker-positive AD vs. biomarker-negative controls in cohort 1. **A**, **B** Boxplots of the concentrations of p-tau231 and p-tau181 in serum and plasma respectively. **C** The diagnostic performance of p-tau231 and p-tau181 in the paired plasma and serum samples, estimated using area under the curve (AUC) calculations

We compared the performance of p-tau231 and p-tau181 when measured in paired serum vs. plasma samples collected from the same blood draw in identical individuals in cohort 1. P-tau231 concentrations ranged from 0.8 to 13.2 pg/ml in serum and 3.3 to 24.4 pg/ml in plasma. When comparing within-individual levels, p-tau231 concentrations were twice higher in plasma (mean = 10.1 pg/ml) vs*.* in serum (mean = 4.6 pg/ml) for the whole cohort (*P*<0.0001). When measured in plasma or serum, mean p-tau231 levels were at least twice higher in biomarker-positive AD vs. biomarker-negative controls (Fig. 1 and Table 1).

For p-tau181, the biomarker ranges were 2.1–18.1 pg/ml in serum vs*.* 3.4–19.1 pg/ml in plasma. Within-individual p-tau181 levels were not statistically different between plasma vs. serum for the whole of cohort 1 despite marginally higher levels in plasma (mean = 7.1 pg/ml for serum and 8.3 pg/ml for plasma, *P =* 0.2333). Serum (mean=9.3 pg/ml vs. 4.5 pg/ml; *P*<0.0001) and plasma (mean=10.4 pg/ml vs. 5.8 pg/ml; *P*=0.0003) p-tau181 were each increased twofold in biomarker-positive AD vs. biomarker-negative controls (Fig. 1 and Table 1). Similar results were found in cohort 2, using the commercial Quanterix kit. P-tau181 concentrations were twice higher in plasma vs. paired serum (mean= 1.1 pg/ml vs. 0.6 pg/ml; *P*<0.0001, Table 1).

In cohort 1, p-tau231 demonstrated high performance to separate the two groups whether measured in serum (AUC=88.2%, 95% CI=75.3–100%) or plasma (AUC=90.2%, 95% CI=79.1–100.0%). For p-tau181, the diagnostic performances were 89.6% (95% CI=78.5–100.0%) in serum and 85.4% (95% CI=71.6–99.2%) in plasma (Fig. 1).

### Validation of the diagnostic value of serum p-tau231 and p-tau181 in an independent cohort

**Fig. 2:**
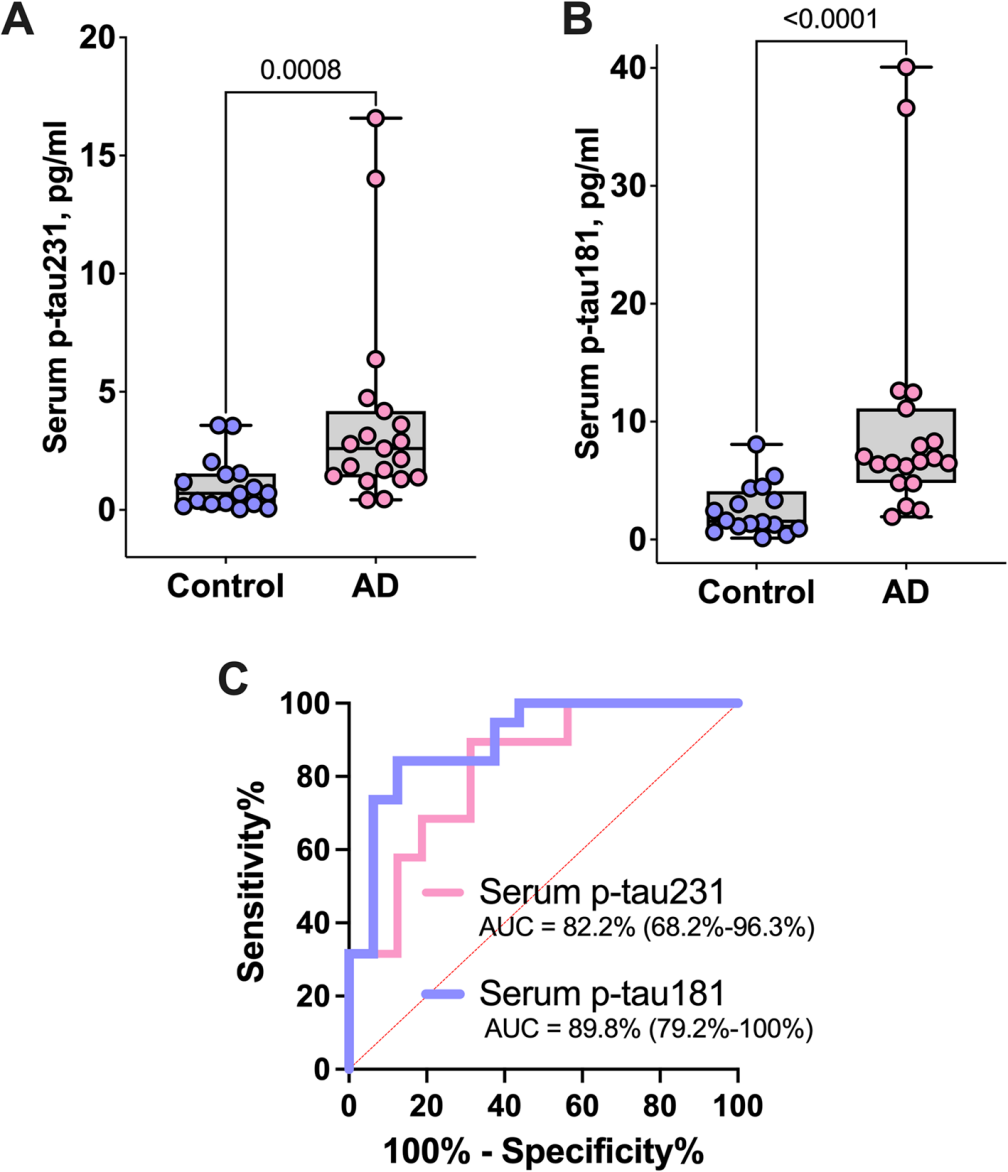
Concentrations and diagnostic performance of serum p-tau231 and p-tau181 in biomarker-positive AD vs. biomarker-negative controls in cohort 3. **A**, **B** Serum p-tau231 and p-tau181 levels respectively in controls and AD, expressed in pg/ml. **C** Area under the curve (AUC) performances of serum p-tau231 and p-tau181 to separate AD from age-matched controls

We further validated the diagnostic performance of serum p-tau in an independent cohort by performing paired measurements of p-tau231 and p-tau181 in both serum and CSF collected at the same clinical visit for each participant in cohort 3 (Table 1). Serum p-tau231 in biomarker-positive AD (mean = 3.8 pg/ml) was three-times higher than in biomarker-negative controls (mean=1.1 pg/ml; *P*=0.0008; Fig. 2A). For p-tau181, a fold change of four was observed in this cohort: mean concentrations of 10.1 pg/ml for biomarker-positive AD and 2.5 pg/ml for biomarker-negative controls (*P*<0.0001; Fig. 2B). Moreover, both serum p-tau231 and p-tau181 differentiated between the two groups with AUCs of 82.2% (95% CI=68.2–96.3%) and 89.8% (95% CI=79.2–100%) respectively (Fig. 2C).

### Results from paired CSF samples suggest brain origin of serum p-tau

To evaluate that the serum results reflect brain-derived p-tau, we compared the p-tau biomarkers in paired serum and CSF samples from identical participants in cohort 3 using the same assay definition on the Simoa technology used for both blood matrices, at the appropriate dilution factors. CSF p-tau231 concentrations in biomarker-positive AD (mean = 777.9 pg/ml) were fivefold higher compared with biomarker-negative controls (mean = 154.4 pg/ml, *P*<0.0001; Table 1). Similarly, CSF p-tau181 was approximately five times increased in biomarker-positive AD (mean = 895.8 pg/ml) vs. in biomarker-negative controls (mean =176.4 pg/ml; *P*<0.0001). Assuming that serum p-tau reflects the same pool of brain-secreted p-tau as in CSF, we estimated the fraction of CSF p-tau released into serum. P-tau231 in serum was a small fraction of paired CSF concentrations without differences in normal aging and disease (0.8% in controls and 0.6% in AD; *P*=0.4814). Despite being similarly small (less than 2%) and reflecting similarly in diseased and control participants, the CSF-to-serum fraction for p-tau181 was double the value recorded for p-tau231 (1.5% for controls vs. 1.5% for AD; *P*=0.4032).

### Associations between blood p-tau and with CSF biomarkers

**Fig. 3:**
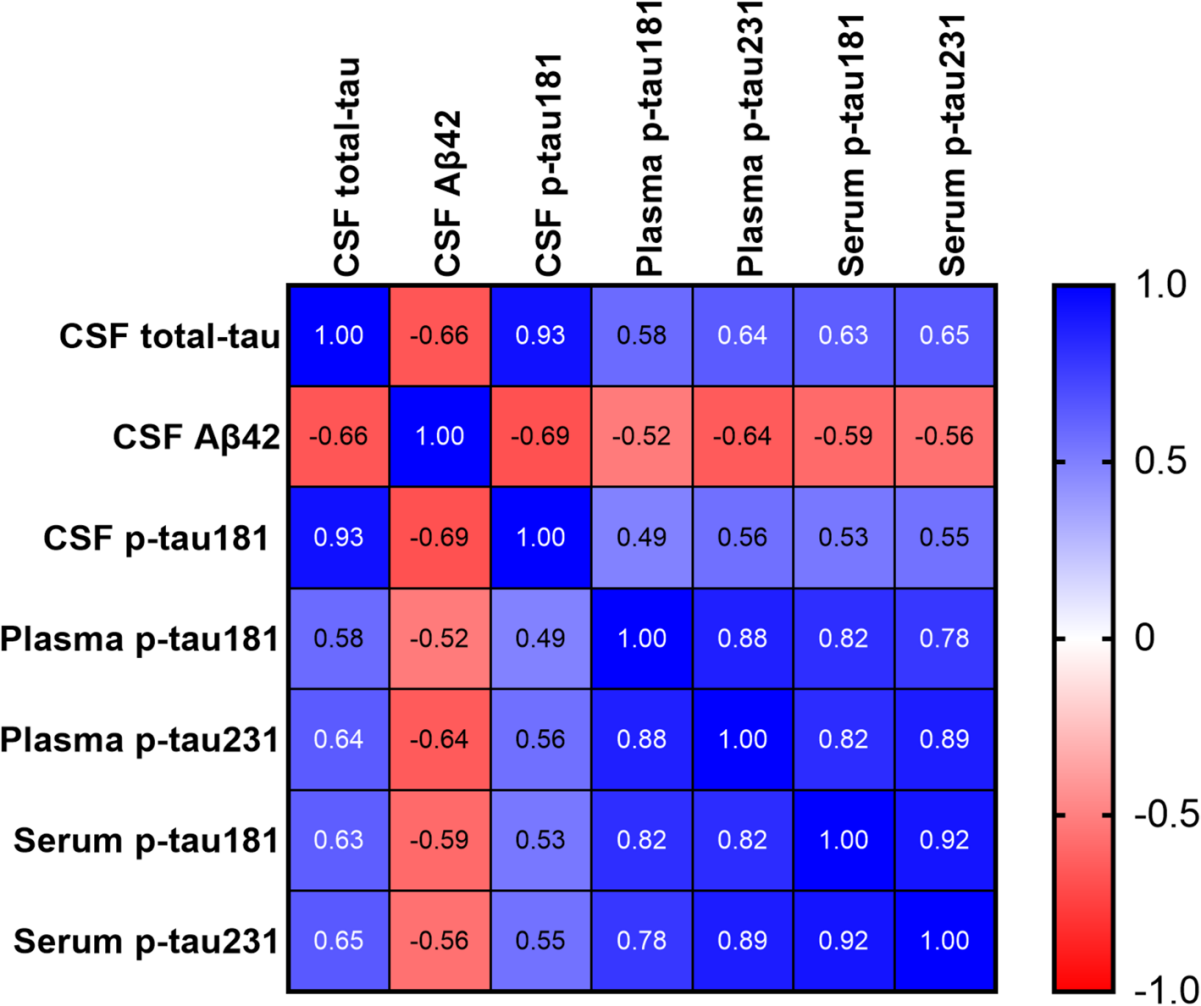
Heatmap of Spearman correlation between blood and CSF biomarkers in cohort 1. The figure shows correlation of plasma and serum p-tau between themselves and with the core CSF biomarkers (Aβ_42_, p-tau181, total-tau) in cohort 1. The heatmap shows a color gradient of −1.0 (red; strongest inverse correlation) to 1.0 (blue; strongest positive correlation)

**Fig. 4:**
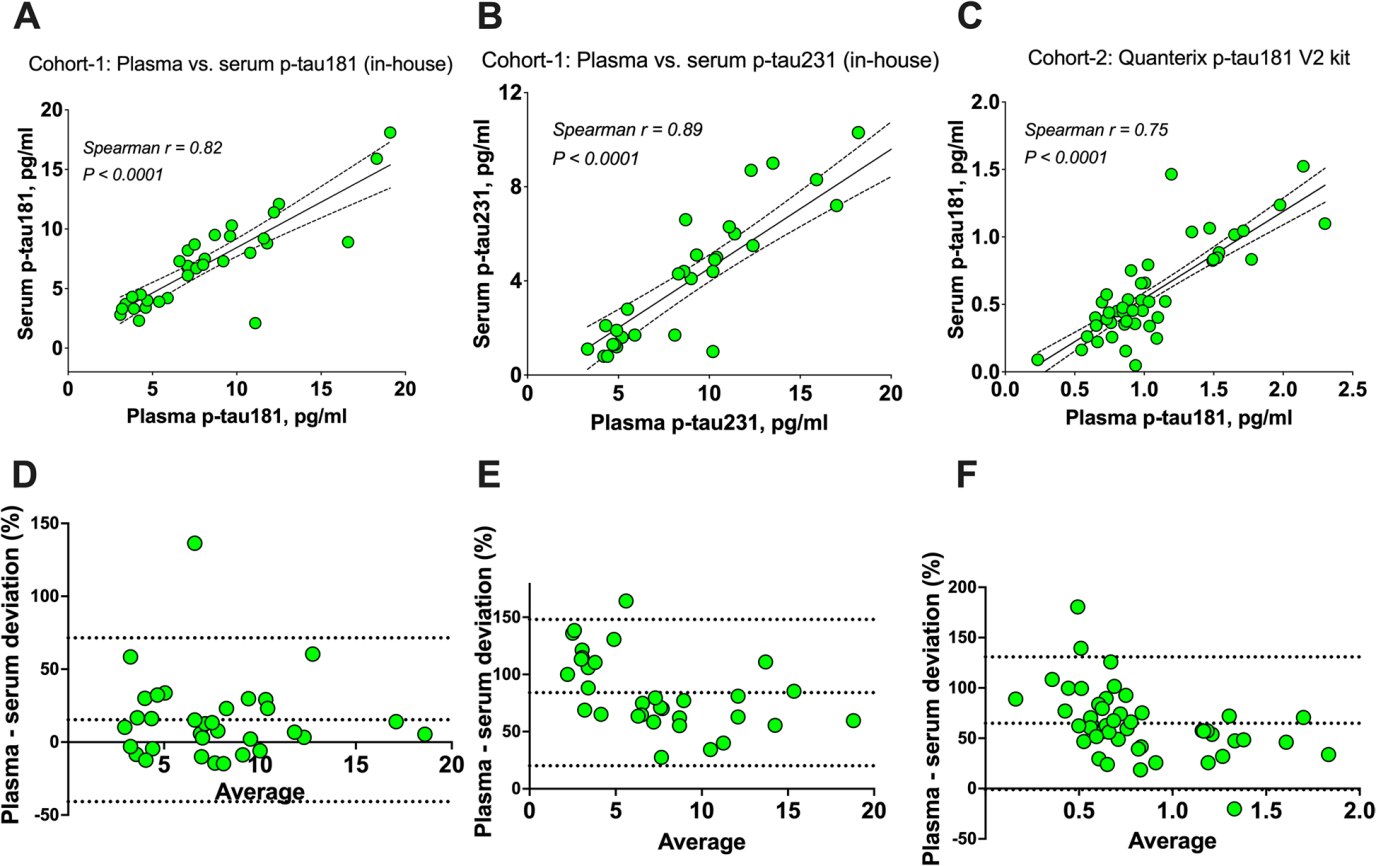
Correlations and biases in paired serum vs*.* plasma p-tau concentrations. **A**–**C** Spearman correlation (rho) of serum vs. plasma p-tau concentrations in paired samples. The plots show correlations for p-tau181 in cohort 1 (**A**), p-tau231 in cohort 1 (**B**), and p-tau181 (using the commercial Quanterix kit) in cohort 2 (**C**). Bland-Altman plots for the same sample pairs are shown in **D**–**F**, respectively. The *y*-axes display the individual differences (serum subtracted from plasma)/average of the two, whereas the *x*-axes display the mean of the two. For each plot, the top and bottom horizontal lines show the 95% limit of agreement while the middle horizontal line shows the estimated bias

In cohort 1 and cohort 2, we recorded significant (*P*≤0.0001) correlations (rho=0.75-0.92) between all blood biomarkers (Figs. 3 and 4). Strong correlations of serum p-tau231 and p-tau181 were observed (rho=0.92, *P*<0.0001; Fig. 3), so were both biomarkers in plasma (rho= 0.88, *P*<0.0001) in cohort 1. For between-matrix correlations for the same biomarker, p-tau231 in serum was strongly correlated with plasma p-tau231 (rho=0.89, *P*<0.0001). Moreover, serum p-tau181 showed a strong correlation with plasma p-tau181 (rho=0.82, *P*<0.0001). Serum p-tau231 correlated with the INNOTEST CSF p-tau181 (rho=0.55; *P*=0.0009) and total-tau (rho=0.65; *P*<0.0001). Serum p-tau181 associated with the INNOTEST CSF p-tau181 (rho=0.53; *P*<0.002) and total-tau (rho= 0.63; *P*<0.0001). Inverse associations were recorded for each of serum p-tau231 (rho= −0.56; *P*=0.0007) and serum p-tau181 (rho= −0.59; *P*=0.0003) vs*.* CSF Aβ_42_. Figure 3 shows Spearman correlations between the blood p-tau markers as well as with the core AD biomarkers in CSF.

In cohort 3, serum p-tau231 and p-tau181 were highly correlated (rho=0.93, *P*<0.0001; Table 2). Moreover, serum p-tau231 showed significant correlations with CSF p-tau231 (rho=0.56; *P*=0.0004), and CSF p-tau181 measured by Simoa (rho=0.57; *P*=0.0003) and INNOTEST (rho=0.55; *P*=0.0006). Serum p-tau231 also correlated negatively with CSF Aβ_42_ (rho= −0.55; *P*=0.0006) and positively with total-tau (rho=0.62; *P*<0.0001). Similarly, serum p-tau181 correlated with CSF p-tau231 (rho=0.70, *P*<0.0001), CSF p-tau181 Simoa (rho=0.71, *P*<0.0001), and INNOTEST p-tau181 (rho= 0.67; *P*<0.0001). Additionally, p-tau181 in serum was associated positively with total-tau (rho= 0.73; *P*<0.0001) and inversely with Aβ_42_ (rho= −0.67; *P*<0.0001) in CSF. Table 2 shows Spearman correlation between each of serum p-tau231 and p-tau181 with CSF biomarkers.

**Table 2:** Spearman correlation of serum p-tau231 and p-tau181 with CSF biomarkers in cohort 3

	Serum p-tau231	Serum p-tau181
CSF Aβ42, pg/ml	rho= -0.55; *P*=0.0006	rho= -0.66; *P*<0.0001
CSF total-tau, pg/ml	rho=0.62; *P*<0.0001	rho= 0.73; *P*<0.0001
CSF p-tau181 (Innotest), pg/ml	rho=0.55; *P*=0.0006	rho= 0.67; *P*<0.0001
CSF p-tau181 (Simoa), pg/ml	rho=0.57; *P*=0.0003	rho= 0.71; *P*<0.0001
CSF p-tau231 (Simoa), pg/ml	rho = 0.56; *P*=0.0004	rho= 0.70; *P*<0.0001
Serum p-tau231, pg/ml	-	rho=0.93, *P*<0.0001
Serum p-tau181, pg/ml	rho=0.93, *P*<0.0001	-

### Equivalence of paired p-tau concentrations in serum vs. plasma

Bland-Altman plots showed mean differences of 15.3% (95% LOA = −40.8 to 71.5%) and 64.9% (95% LOA = −1.1 to 130.8%) for the serum-plasma p-tau181 pairs in cohort 1 and cohort 2 respectively. Similar results were obtained for p-tau231 in cohort 1: mean difference of 84%, 95% LOA = 20.1–148.0% (Fig. 4). Overall, the relative difference was highest in the lower concentration range of p-tau231 in cohort 1, and p-tau181 in cohort 2.

## Discussion

The results of this pilot study showed that serum may be a viable blood matrix for the assessment of the novel p-tau231 AD biomarker that has recently been verified for use in plasma [5, 9, 22, 23, 34]. Increases in serum p-tau231 discriminated between biomarker-positive AD vs. biomarker-negative controls with AUCs of up to 88.2%, equivalent to 90.2% for plasma. Moreover, we extended earlier results that plasma p-tau181 is measurable in serum [1, 24, 26, 29], demonstrating that diagnostic performance is similarly high when measured in serum or plasma (89.6% and 89.8% vs. 85.4%). We further validated the utility of serum p-tau231 and p-tau181 by showing (i) similar correlations vs. the same biomarkers measured in paired plasma samples and (ii) significant correlations with biomarkers measured in CSF samples from the same individuals. Together, the results suggest that p-tau biomarkers in serum reflect brain pathophysiological changes and may be employed to support clinical and research-based evaluation of AD. Nonetheless, we recorded biases of 15–84% between the absolute concentrations of paired p-tau measures to suggest that biomarker levels are not interchangeable between serum vs plasma. Furthermore, the consistently lower absolute concentrations of p-tau231 in serum compared with plasma may pose analytical challenges that need addressing especially for Aβ-negative individuals, as illustrated in the lower concentration ranges, where the relative differences between the two matrices were larger (Fig. 4D–F).

Blood p-tau biomarkers have been validated in EDTA plasma to show excellent diagnostic and analytical performances [1, 2, 5, 22, 23, 34, 38], but their utility in other blood matrices is unclear. In the present study, we have demonstrated that the biomarker performances of plasma p-tau231 and p-tau181 are replicable in serum, expanding the repertoire of blood matrix types that are suitable for evaluating these biomarkers. Additionally, p-tau231 and p-tau181 correlated strongly with each other when measured in serum, plasma, and CSF, demonstrating corresponding elevations of both biomarkers in AD that are quantifiable in multiple bodily fluids [5, 10, 31]. Notably, there were similar correlations of p-tau231 and p-tau181 when both were measured in serum (rho = 0.92) vs*.* in plasma (rho = 0.88) in cohort 1. Additionally, the serum-based correlation of p-tau231 and p-tau181 in cohort 3 (rho = 0.93) was similarly as high as in cohort 1. The serum biomarker correlations in each cohort were stronger than previous results in plasma (rho = 0.6) [5].

While there were strong correlations between paired serum and plasma p-tau levels and either modality accurately differentiated between AD and controls, Bland-Altman plots support a bias in serum vs. plasma concentrations in paired samples, with the disagreements being higher at lower average values. This is explained by the observed differences in absolute levels of p-tau measures in paired samples from the same individuals. For example, paired p-tau231 levels were twofold higher in plasma compared with serum in cohort 1. Similarly, p-tau181 levels were higher in plasma vs*.* serum in both cohorts 1 and 2, although these differences reached statistical significance only in cohort 2. These consistently lower p-tau concentrations in serum vs*.* in plasma are in agreement with recent reports for p-tau181 and total-tau [1, 24, 26]. Thus, we propose that the measured concentrations of plasma and serum p-tau biomarkers are not interchangeable despite values in either matrix showing strong correlations and excellent diagnostic performances. In effect, it is important to use either plasma or serum samples independently for an entire study, including longitudinal monitoring, without switching between matrix types.

Translation of our results that serum is equally good for blood-based p-tau analyses as plasma will require careful standardization of how whole blood is processed into serum. At present, the standard operation procedures for serum generation are somewhat vague and are likely to vary between hospital systems and research institutions. For instance, most manufacturers recommend that whole blood should be incubated at room temperature for 30 min to 1 h to form clots that separate the liquid fraction from cellular components. Deviating from these recommendations can have consequences. For instance, removal of cellular elements from the liquid portion is less complete in samples incubated for less than 30 min [39]. Additionally, samples incubated or transported for over 60 min tend to experience cell lysis to release cellular materials that are not usually found in serum [39]. For patients or research participants known to be on anticoagulant treatments, longer incubation period may be necessary for clot formation. Standardization of preanalytical factors (e.g., time of clotting, centrifugation, and aliquoting) between and within studies is important to ensure reproducibility of p-tau results collected using serum samples.

Furthermore, p-tau measurements in serum are likely to result in more values below the lower limit of quantification of several p-tau assays given the lower biomarker concentrations in this matrix versus EDTA-plasma. Potential disadvantages include challenges in differentiating cognitively normal individuals with preclinical evidence of AD from those without since the biomarker levels between these groups tend to be marginally different [19].

Assuming that p-tau secreted to serum is a fraction of the same pool of p-tau molecules that are secreted to the CSF from the brain, we estimated that <1% of CSF p-tau231 concentrations reflect in serum, with comparable results found for p-tau181 (<2%) in cohort 2. The similarities in CSF-to-serum fractions between controls and AD suggest that p-tau transport/release between CSF and blood in normal aging remains unchanged in AD. These results are also in agreement with our previous finding of 5% CSF to plasma fraction [1].

CSF Aβ_42_/Aβ_40_ is an early AD biomarker, often becoming abnormal ahead of Aβ-PET [40]. The accessibility, cost-effectiveness, and simplicity advantages of blood make it a highly attractive biofluid for clinical chemistry evaluation of biological changes in disease. Hence, identifying a blood-based biomarker that performs closely to CSF Aβ_42_/Aβ_40_ for brain amyloidosis is crucial to enable large-scale screening in population and epidemiological studies for potential preclinical AD candidates for inclusion in anti-amyloid therapeutic trials. This is made even more relevant and urgent by the recent approval of the anti-amyloid drug Aduhelm® and the ongoing consideration of other promising candidate drugs by the FDA [41]. However, measuring Aβ in blood is analytically challenging due to small dynamic ranges even when measured with immunoprecipitation-mass spectrometry (IPMS) assays [19]. This challenge is even more acute in serum where concentrations are much lower, correlate poorly with plasma Aβ, and are sensitive to freeze-thaw cycling [24, 26]. It has therefore been recently recommended that serum be avoided for blood Aβ measurements due to unreliable results [24]. Even in plasma, some immunoassay methods have poor performance while IPMS techniques that have superior diagnostic utility are time- and resource-intensive [18, 42]. While plasma IPMS Aβ can sometimes detect amyloidosis equally or slightly better than plasma p-tau particularly in preclinical AD [7], current technical difficulties limit its throughput and widespread adoption [17, 18]. Moreover, plasma p-tau217, which has shown substantial potential for brain amyloidosis in blood and CSF [2, 10, 31, 43], is currently only reliably measurable in plasma; quantification in serum looks less promising given very low concentrations even in plasma, often below the detection limit [2, 44–46]. Since plasma p-tau biomarker levels in preclinical AD are only marginally increased compared with biomarker-negative controls [1, 2, 6–8, 10, 11, 32], robust and reproducible measures are essential. Together, there is presently a limited toolbox of accessible blood biomarkers to screen for and to longitudinally monitor preclinical AD participants undergoing clinical trials. To this end, we reported that plasma p-tau231 starts to increase in the “pre-amyloid phase” before Aβ-PET abnormality thresholds are reached [5]. Furthermore, plasma p-tau231 outperformed plasma p-tau181 and CSF p-tau217 for preclinical AD, for which reason plasma p-tau181 performed better at separating AD dementia and Aβ+ cognitively unimpaired elderly [5]. The slightly lower diagnostic accuracy of serum p-tau231 for AD vs*.* controls compared with p-tau181 in the present study seems to replicate the preclinical capacity of p-tau231 in brain, CSF, and plasma [5, 10, 31, 47], since the biomarker-negative control group most likely included those with emerging AD pathology. A significant finding in the present study is that by showing that p-tau231 has good performance in serum that match its demonstrated performance as an early amyloid marker in plasma, we show that the field can expand blood p-tau analyses to include matrices like serum which is preferred in some medical centers and clinical studies.

Major strengths of this study include the evaluation of p-tau231 and p-tau181 in paired plasma and serum (cohorts 1 and 2) as well as paired serum and CSF (cohort 3) samples, providing insights into p-tau levels in the central nervous system and the periphery. Moreover, we used two variations of the same p-tau181 method, the in-house Gothenburg p-tau181 assay and its commercially adapted variant available from Quanterix, meaning that the results obtained herein can be verified in other cohorts or research settings with access to the commercial Simoa method.

### Limitations

The study had a decent sample size, given inherent difficulties to obtain paired samples provided at the same patient visit. Future studies should verify the results in larger cohorts and determine any relevant diagnostic advantages or disadvantages between plasma and serum. Regrettably, the small sample size and a lack of CSF Aβ_42_/Aβ_40_ or Aβ-PET data prevented in-depth analyses of the diagnostic performance of serum p-tau231 in preclinical AD.

## Conclusion

Serum p-tau231 and p-tau181 showed good performances for diagnostic and research purposes but the former was at lower concentrations than what was observed for the same biomarker in paired plasma samples. Significant increases of serum p-tau231 and p-tau181 in biomarker-positive AD vs*.* biomarker-negative controls and the strong intra- and inter-matrix correlations between themselves and with other biomarkers agree with previous results in plasma and CSF, authenticating feasibility of p-tau assessments in serum. Together, our results suggest that serum is a viable matrix for p-tau analysis, for both p-tau181 and p-tau231, providing a practical alternative to plasma especially in hospitals and research cohorts that prefer serum to other blood matrices. These results should be validated in independent cohorts and for different p-tau assays.

## Data Availability

All data generated or analyzed during this study are included in this published article.

## Abbreviations

AD: Alzheimer’s disease
CSF: Cerebrospinal fluid
EDTA: Ethylenediaminetetraacetic acid
LOA: Limit of agreement
P-tau: Phosphorylated tau
USFDA: United States Food and Drug Administration
